# Strawberry Protease as a Laundry Detergent Additive Candidate: Immobilization, Compatibility Study with Detergent Ingredients, and Washing Performance Test

**DOI:** 10.1002/gch2.202300102

**Published:** 2023-11-24

**Authors:** Esma Hande Alici, Gulnur Arabaci

**Affiliations:** ^1^ Department of Chemistry Faculty of Science Sakarya University Serdivan‐Sakarya 54187 Turkey

**Keywords:** detergents, *fragaria ananassa*, immobilization, protease, strawberry

## Abstract

The potential of strawberry‐derived protease as a component of laundry detergent is investigated. The compatibility of the enzyme with various surfactants, oxidizing agents, and commercial detergents is tested. The immobilized enzyme prepared by immobilizing Co^2+^ ions together with the enzyme is also tested. Strawberry crude protease shows high stability in the presence of surfactants frequently used in detergents. The enzyme is found to be relatively stable to oxidizing agents. In addition, it is determined that strawberry protease works in excellent compatibility with different commercial solid and liquid detergents in the Turkish market and also maintains its stability very well. Washing tests based on visual examination also reveal that the enzyme improves the washing performance of the tested detergent. All these properties and high activity at alkaline pH make this enzyme a very strong candidate for use in laundry detergent formulations.

## Graphical abstract

Strawberry crude protease has great potential as a laundry detergent additive. Protease works harmoniously with the tested detergent components. Protease shows excellent stability in the presence of commercial detergents. Co^2+^ ions increase the stain removal contribution of the protease. Immobilization enhances the thermal/pH stability of the enzyme.

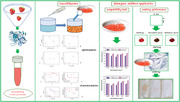

## 1 Introduction

Enzymatic biocatalysis plays a significant role in green chemistry due to the specificity and high catalytic efficiency of enzymes. With today's world being exposed to numerous pollutants and toxic processes, there is a shift toward environmentally friendly products, resulting in a greater number of chemical procedures being replaced with enzymatic methods. As the demand for useful biocatalysts increases, the world enzyme market is also growing and the number of studies on enzyme production and applications is increasing rapidly.^[^ ^1^, ^2^ ^]^ Most of the enzymes used in industry are hydrolytic and are used in many degradation processes.^[^ ^2^ ^]^ Proteases, which are hydrolytic enzymes that enable the degradation of proteins into small peptides and amino acids, have become the most frequently used group of enzymes in the industry today due to both being an environmentally friendly material and a wide range of their useful applications.^[^ ^3^, ^4^ ^]^ In terms of commercial enzyme sales, proteases constitute the largest product segment in the market.^[^ ^5^, ^6^ ^]^ The marketing value of global enzymes in 2021 is reported to be $11.47 billion, and this value is estimated to increase further in the near future. Proteases rank first among all enzymes in this market.^[^ ^7^ ^]^ Protease enzymes are frequently used in the food, pharmaceutical, textile, and leather industries and, particularly, in the detergent industry where alkaline proteases are used. Likewise, they possess specific uses in biotransformation reactions, waste management, peptide synthesis, extracting silver from X‐ray/photo films, agriculture, feed additives, and as an active agent in various medicinal therapies.^[^ ^2^, ^8^, ^9^, ^10^, ^11^ ^]^ When focusing on the use of proteases in the detergent industry, it is seen that this enzyme is beyond being just an additive in detergent production and is one of the basic components in the formulation of all kinds of detergents (laundry, dishwashing, industrial cleaning) today.^[^ ^2^, ^10^, ^12^ ^]^ Proteases in the detergent composition improve the cleaning capacity and increase the efficiency of detergents by acting on protein‐based stains such as food stains, blood, and grass.^[^ ^5^, ^11^ ^]^ Detergent formulations often contain surfactants, bleaches, enzymes, and some additives. Surfactants are important in detergent formulations as they play a major role in cleaning performance.^[^ ^13^ ^]^ The foaming and cleaning abilities of liquid detergents, which are made from a combination of surfactants used especially in household laundry, are being improved day by day with successful studies.^[^ ^14^ ^]^ Therefore, the enzymes included in the detergent composition must be compatible with all detergent components, especially surfactants. The expectations from a protease to be used in a detergent composition are defined by many parameters such as optimum activity and stability over a wide temperature range and especially at alkaline pH values, compatibility with other detergent components, as well as stability in the presence of oxidizing agents.^[^ ^5^, ^10^ ^]^ Enzymes intended for use in industrial processes are expected to have some properties such as stability over a range of temperature and pH, and show their capability to work in the presence of various chemical substances.^[^ ^15^ ^]^ Since industrial processes require large‐scale production and low cost, microbial enzymes have replaced plant‐based industrial enzymes over time, but these expectations also necessitate the discovery of effective and stable new protease enzymes.^[^ ^2^, ^6^, ^9^, ^10^ ^]^ Accordingly, new proteases should be investigated by isolating these enzymes from different sources and characterizing them under targeted conditions.^[^ ^11^, ^12^ ^]^ As a result, plant‐derived enzymes, which have attracted industrial attention for their unique catalytic properties and stability over a wide pH and temperature range, are also becoming a suitable source of proteases for the detergent industry.^[^ ^16^, ^17^ ^]^ Strawberry protease enzyme is a promising candidate as a detergent component, with properties such as broad substrate specificity, high activity at alkaline pH, and thermostability over a wide temperature range, as well as widespread distribution and ease of cultivation of its source.^[^ ^18^ ^]^ In brief, this study aims to investigate the potential of strawberry proteolytic extracts as a component that can improve the cleaning performance of laundry detergents. Within the scope of the study, the strawberry protease was first immobilized to ensure reusability, which is of great importance for industrial processes, and after the characterization of the immobilized enzyme, its compatibility and stability with various detergent components/commercial detergents were tested, and finally, the effect of the strawberry protease enzyme on the cleaning efficiency of commercial detergent was investigated.

## 2 Results and Discussion

### 2.1 Immobilization of Strawberry Protease

#### 2.1.1 Optimization of Immobilization Conditions

Different concentrations of sodium alginate (Na‐alginate), calcium chloride, and crude enzyme solution were tried during the preparation of the beads to obtain more stable beads. To determine more measurable activity values, after determining the optimum concentrations of the components used in immobilization, the immobilized enzyme beads obtained from the immobilization process under optimum conditions were used in different amounts in the reaction medium (3–11 beads; 0.1–0.4 g), and the specific activity results were compared. The results of the optimization tests are given in Figure S1, Supporting Information. According to the evaluation of the specific activity results of the immobilized enzymes produced under different conditions, the highest enzyme activity was observed when using 2% w/v Na‐alginate concentration, 3% w/v calcium chloride concentration, fourfold diluted crude enzyme (DF = 4; 10 mg mL^−1^), and 0.4 g of immobilized beads. Therefore, immobilization of strawberry protease was carried out using these values.

The pore size of the beads should be wide enough for the substrate and the product to easily diffuse into and out of the alginate gel matrix that holds the enzyme in its microenvironment. As the concentration of the Na‐alginate solution decreases, the pore size of the beads increases. This results in a higher amount of enzyme leaching through the beads. Conversely, higher concentrations of Na‐alginate result in smaller pore sizes in the beads, causing a decrease in immobilization efficiency.^[^ ^19^ ^]^ Leakage of enzymes from the formed beads was observed more at 1% w/v Na‐alginate concentration due to the larger pore size. The activity of the entrapped enzyme was found to be relatively lower at 3% w/v and 4% w/v Na‐alginate concentrations. This may be due to the high viscosity of the beads, which results in a smaller pore size, making it more difficult for the substrate to penetrate the interior of the enzyme. The results of various studies are available in the literature, indicating that a Na‐alginate concentration of 2–3% w/v is more suitable for the immobilization of various enzymes such as keratinase, lipase, and proteases.^[^ ^20^, ^21^, ^22^ ^]^ Like strawberry protease, Na‐alginate concentrations in the range of 1–4% w/v were tested in optimization studies for *Bacillus subtilis* KIBGE‐HAS protease immobilized in Ca‐alginate beads. The results of the alginate concentration optimization of this protease were generally similar to strawberry protease, and the optimum alginate concentration of the enzyme was found to be 2% w/v.^[^ ^23^ ^]^

The highest specific activity value for the strawberry protease was obtained at 3% w/v calcium chloride concentration; besides, the result of 4% w/v calcium chloride solution was almost the same as that of 3% w/v. The specific activity increased with increasing percentage of calcium chloride up to a concentration of 3% w/v. In the immobilization of Capricorn dipeptidyl peptidase IV enzyme in Ca‐alginate beads, optimum activity was reached at 1% w/v CaCl_2_, while the activity of the immobilized enzyme gradually decreased when this value was exceeded.^[^ ^24^ ^]^

Araujiain, a cysteine protease, was immobilized by entrapment in Ca‐alginate beads. During the optimization of the immobilization conditions, different amounts of enzyme (0.5–25 mg) were immobilized in the beads and how the activity changed was investigated. According to the results, enzyme activity remained at a very low level up to 10 mg enzyme; however, there was a significant increase in enzyme activity around 10–25 mg.^[^ ^25^ ^]^

Cells of *Teredinobacter turnirae*, a marine bacterium, were immobilized in Ca‐alginate beads and used for alkaline protease production. While optimizing the immobilization conditions, the number of immobilized beads used was also changed and tried to be brought to the optimum level. For this purpose, enzyme activity was determined by using 10–300 beads in the reaction medium. Enzyme activity increased significantly as the number of beads increased to a total of 200 beads. Above this number of beads, no significant increase in enzyme activity was observed. Thus, experiments were carried out using 200 immobilized beads in the remainder of the study.^[^ ^22^ ^]^

The difference between the optimization results was also statistically evaluated by a one‐way ANOVA test, and the *p* values were found to be <0.0001 for all four parameters tested. Then, a further evaluation was made with the Bonferroni post hoc test. According to Bonferroni test results, there was a significant difference in each of the results obtained at different alginate concentrations compared to the other. An increase in the specific activity of the protease up to 3% (w/v) calcium chloride concentration was significant, and there was no significant difference between 3% w/v and 4% w/v. While there was no significant difference between the results obtained for enzyme dilution factors 2 and 3, the difference between the results of the other dilution factors was found to be statistically significant. It was also found that the increase in specific activity due to the increase in the number of beads used was significant after 0.2 g.

#### 2.1.2 Investigation of the Co^2+^ Ions’ Effect on Immobilization

During the immobilization of the protease carried out under optimum conditions, Co^2+^ ions were also immobilized with the enzyme in the beads formed. The reason for choosing Co^2+^ ions is that these ions showed a strong activator feature for the strawberry protease in our previous study.^[^ ^18^ ^]^

In this study, it was aimed to increase the efficiency of the immobilized enzyme with the Co^2+^ ions. There are other studies in the literature on metal ions that strongly activate proteases. Similarly, Gaur and Wadhwa stated that an alkaline protease from *Lantana camara* was strongly activated by Co^2+^ ions.^[^ ^26^ ^]^ It was reported that the in vitro proteolytic activity of the protease called acrosin in pig sperm was stimulated by several monovalent and divalent metal ions. Divalent metal ions caused a 24‐fold (magnesium) and 46‐fold (calcium) increase in proteolytic activity, while equivalent concentrations of monovalent cations increased the proteolytic activity by two to three times.^[^ ^27^ ^]^

The activities of the immobilized enzymes containing Co^2+^ at different concentrations (1, 2, and 5 mm) were compared with the activity of the immobilized enzyme without Co^2+^, and the results are given in Figure S2, Supporting Information. Considering the specific activity results, it was determined that the Co^2+^ ions immobilized with the enzyme significantly increased the enzyme activity (*p* < 0.0001 [one‐way ANOVA test], and according to the Bonferroni test, the change in activity was significant compared to control for all tested Co^2+^ ion concentrations). The highest stimulation was observed when Co^2+^ ions were used at a 1 mm concentration level. The enzyme activity showed a large increase compared to the control at 1 and 2 mm; however, a decrease in activity occurred at 5 mM. This may be due to the fact that the area in the beads surrounding the enzyme was filled with large amounts of Co^2+^ ions, and consequently, substrate and product diffusion became difficult. As a result of the experiment, an 8.4‐fold increase in activity at a 1 mm Co^2+^ level, a 5.1‐fold increase in activity at a 2 mm Co^2+^ level, and a 29% decrease in activity at a 5 mm Co^2+^ level were observed compared to the control.

#### 2.1.3 Evaluation of the Immobilization Success

In gel beads, the concentration of Na‐alginate and CaCl_2_ are critical parameters for enzyme entrapment, since the factor that ensures gel formation is crosslinking between alginate and Ca^2+^ ions. Therefore, various concentrations of Na‐alginate and CaCl_2_ were tried to obtain the maximum “immobilization yield.” As can be seen from Table S1, Supporting Information, the highest immobilization activity yield was obtained at 2% w/v alginate concentration and 3% w/v CaCl_2_ concentration. Consequently, while immobilizing strawberry protease, bead formation was studied at these two constant concentrations. Accordingly, the immobilization yield was found to be 65% when only the crude enzyme was immobilized in beads, and 69% when the crude enzyme was immobilized together with Co^2+^ ions. Since Co^2+^ ions activated strawberry protease, immobilization yield increased in the presence of Co^2+^ ions (the increase in yield was found to be insignificant, *p* > 0.05 [one‐way ANOVA test]).

While the efficiency value calculated for the immobilized crude protease was 63%, it increased to 89% by the immobilization of the Co^2+^ ions with the crude protease (the increase in efficiency was statistically significant, *p* < 0.05 [one‐way ANOVA test]). Similarly, activity recovery increased from 41% to 61% with the immobilization of Co^2+^ ions with crude protease (Table S1, Supporting Information). The activity recovery value of 41% indicated an apparent decrease in the initial activity with immobilization of the enzyme in the beads. However, this decrease was significantly eliminated by immobilizing Co^2+^ ions with the enzyme (the increase in activity recovery was statistically significant, *p* < 0.05 [one‐way ANOVA test]). Since Co^2+^ ions were used at a low concentration of 1 mm, they did not pose a major obstacle to substrate/product diffusion but had a positive effect on the success of the immobilization process by significantly increasing enzyme activity. In view of this information, it can be said that strawberry protease was successfully immobilized even without Co^2+^ ions. The presence of Co^2+^ ions increased the immobilization success in the expected direction. In the study in which *B. subtilis* MTCC 10422 milk‐precipitating protease enzyme was immobilized in the alginate‐pectate gel by entrapment method, the immobilization yield was 97.78 ± 4.05%; immobilization efficiency was 74 ± 0.02% and activity recovery was 73 ± 0.04%.^[^ ^28^ ^]^

### 2.2 Characterization Results

#### 2.2.1 pH Profile

**Figure 1:**
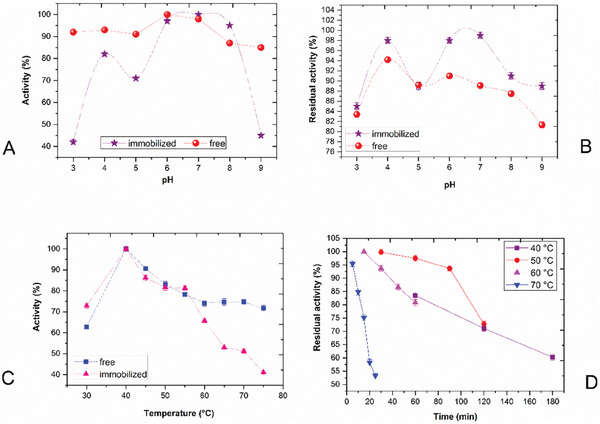
Thermal and pH profile of immobilized and free strawberry protease enzyme. Error bars represent the standard deviation of triple measurements. One‐way ANOVA test was used for statistical evaluation. A) Optimum pH graph of immobilized and free protease. *n*: 14; after immobilization, the change in activity was significant (*p* < 0.05) compared to free enzyme at acidic and basic pH values. B) pH stability graph of immobilized and free protease. *n*: 14; after immobilization, the increase in stability was not statistically significant (*p* > 0.05) compared to free enzyme. C) Optimum temperature graph of immobilized and free protease. *n*: 18; after immobilization, the change in activity was found to be significant (*p* < 0.05) when the temperature value was 60 °C and above compared to free enzyme. D) Thermal stability graph of immobilized protease. *n*: 32; a significant increase was observed in the thermal stability, above 40 °C (*p* < 0.03).

To determine the pH value at which the immobilized protease enzyme showed optimum activity, activity measurements were carried out at different pH values between pH 3 and 9, and these activity values are given as relative activity (%) in **Figure** **1**A. The activity change of the free enzyme depending on the medium pH was also given in the same graph. The maximum activity for the immobilized enzyme was obtained at pH 7. In other words, it was observed that the optimum pH value for strawberry protease shifted from pH 6 to 7 as a result of immobilization. However, it should be noted that the activity values at pH 6 and 7 were very close to each other for both free and immobilized enzymes. Therefore, the optimum pH shift indicated here was not a significant change.

However, the relative activity results also differed when compared to the free protease. While activity values close to each other were obtained at all pH values for free protease, a significant decrease in enzyme activity was observed at acidic and basic pH values compared to neutral pH values as a result of immobilization (*p* < 0.05 [one‐way ANOVA test]; after immobilization, the change in activity was significant compared to free enzyme at acidic and basic pH values). The anionic or cationic nature of the support surface and the microenvironment of the immobilized enzyme can change the optimum pH of the enzyme by up to 2 pH units. This may be accompanied by a general broadening of the pH region in which the enzyme can work effectively.^[^ ^29^ ^]^ Accordingly, the interactions between the charged groups on the bead surface and the enzyme structure may have caused these changes. Similarly, immobilization of another serine protease, dipeptidyl peptidase IV (DPP IV), in Ca‐alginate beads, was found to shift the pH optimum by 0.5 units towards the basic side.^[^ ^24^ ^]^ In another study, it was reported that the optimum pH value of Araujiain, a cysteine protease entrapped in Ca‐alginate beads, shifted from 8.5 to 8 with immobilization.^[^ ^25^ ^]^

In the stability studies of the immobilized enzyme, it was observed that the enzyme retained most of its activity at all pH values when incubated for 3 h with buffer solutions with pH values between 3 and 9 (Figure 1B). At the end of the incubation period, the highest activity loss was calculated as 15% at pH 3 compared to the control. According to the results, the stability of the immobilized strawberry protease in a wide pH range was quite high.

The stability of immobilized enzymes may increase or decrease depending on the type of carrier and the interaction between the enzyme and the carrier. During immobilization, parameters such as immobilization matrix, immobilization conditions, and solidification by multi‐point bonding are important determinants of enzyme stability.^[^ ^30^ ^]^ When the pH stability results of the immobilized enzyme were examined, it was detected that the stability increased at all pH values compared to the free enzyme. As a result, it can be said that the immobilization process increased the stability of the enzyme (*p* > 0.05 [one‐way ANOVA test]; after immobilization, the increase in stability was not statistically significant compared to free enzyme; Figure 1B). Similar to strawberry protease, an increase in stability of Araujiain cysteine protease at all pH values tested was determined after immobilization in Ca‐alginate beads.^[^ ^25^ ^]^ Unlike strawberry protease, the stability of DPP IV serine protease at neutral pH values did not change after entrapment in Ca‐alginate beads, while its stability at acidic and basic pH values increased. Furthermore, this increase was not as pronounced as in strawberry protease.^[^ ^24^ ^]^

#### 2.2.2 Thermal Profile

Temperatures between 30 and 75 °C were tested to determine the temperature at which the immobilized protease showed optimum activity, and the maximum activity was obtained at 40 °C (Figure 1C). In brief, when strawberry protease was immobilized, there was no change in the optimum temperature value. Marathe et al., in their study, used *B. subtilis*, *Pseudomonas aeruginosa*, and *Alcaligenes faecalis* bacteria isolated from seawater in the production of alkaline proteases. Then, they immobilized each enzyme separately in Ca‐alginate beads and characterized both free and immobilized enzymes. According to the results, the optimum temperature values of free alkaline protease enzymes obtained from *B. subtilis*, *P. aeruginosa*, and *A. faecalis* bacteria were determined as 55, 50, and 55 °C, respectively. After immobilization, the optimum temperature values of *P. aeruginosa* and *A. faecalis* alkaline proteases did not change, while the optimum temperature value of *B. subtilis* alkaline protease shifted from 55 to 60 °C.^[^ ^31^ ^]^

The immobilized strawberry protease was incubated at temperatures between 40 and 70 °C with the purpose of determining the thermal stability. It was determined that, at 40 °C, the activity of the immobilized enzyme was almost not affected after 1 h of incubation, a slight decrease in its activity was observed after 2 h of incubation, and still retained 60% of its activity after 3 h. Besides, at 50 °C, it was detected that the enzyme retained most of its activity after 90 min and lost only 28% of its activity after 120 min. While there was no decrease in enzyme activity at the end of the 15th min at 60 °C, it was found that the enzyme lost only 20% of its activity at the end of the 60‐min incubation period. Finally, at a temperature as high as 70 °C, it is an important finding that the enzyme retained 75% of its activity until the end of the 15th min and still retained half of its activity at the end of the 30 min (Figure 1D).

Immobilization may significantly influence the stability of an enzyme. If the immobilization process causes rigidity in the enzyme, this tends to promote the inactivation of the enzymes under denaturing conditions (e.g., high pH/temperature values). However, tremendous stability can occur when there is some kind of unstretched multi‐point bonding between enzyme and support. This is owing to the physical prevention of major structural changes in the basic protein structure.^[^ ^32^ ^]^ As a result of the immobilization of strawberry protease by the entrapment method, a significant increase was observed in the thermal stability (above 40 °C, *p* < 0.03, one‐way ANOVA). This increase in stability became even more pronounced with longer incubation times, especially at the related temperatures. For instance, after 30 min of incubation at 50 °C, 92.23% of free protease activity and 99.77% of immobilized protease activity were retained. The difference was not very considerable. However, the stability measurements obtained for the immobilized protease at the end of the incubation periods of 60, 90, and 120 min at the same temperature were considerably higher than the measurements obtained for the free protease, and the difference became wider as the time increased. In a study in which a milk‐precipitating protease purified from *B. subtilis* MTCC 10422 was immobilized in an alginate‐pectate gel, the thermal stability of the free and immobilized enzyme in the temperature range of 40–70 °C was investigated in detail. According to the findings, similar to strawberry protease, the enzyme became more stable thermally as a result of immobilization, and the increase in stability became evident as the temperature value increased.^[^ ^28^ ^]^

#### 2.2.3 Kinetic Parameters of BSA Hydrolysis

In the experiment performed with immobilized crude protease, initial velocity values were measured using substrate solutions in the concentration range of 0.015 and 0.38 mm (15.2–380 µm) to determine the kinetic parameters of BSA hydrolysis. A Lineweaver–Burk diagram was created using the measured initial velocities, and kinetic constants were calculated using this diagram. To accurately determine the change of kinetic constants with immobilization, the Km and Vmax values of the free crude enzyme were determined under the same conditions (Figure S3, Supporting Information). The Km and Vmax values of BSA hydrolysis at 40 °C and pH 6 were found to be 0.041 mm and 2.48 µmol tyrosine mL^−1^ min^−1^ for free crude protease, and 0.089 mm and 2.47 µmol tyrosine mL^−1^ min^−1^ for immobilized crude protease, respectively. A relatively insignificant decrease (*p* > 0.05, one‐way ANOVA) in Vmax was accompanied by a significant increase (*p* < 0.0001, one‐way ANOVA) in Km. Accordingly, the increase in Km as a result of immobilization can be interpreted as a decrease in the enzyme's affinity for the BSA substrate. In general, the kinetic behavior of an immobilized enzyme can differ significantly from that of the same enzyme in a free solution. While the properties of an enzyme can be modified by the selection of the appropriate immobilization protocol, the same method can have significantly different effects on different enzymes. These changes may be due to conformational changes in the enzyme depending on the immobilization procedure or the availability and nature of the support used in the immobilization.^[^ ^32^ ^]^ The increase in the Km value of crude strawberry protease for the BSA substrate may be largely due to diffusion problems. Likewise, because of the immobilization in Ca‐alginate beads of two separate proteases partially purified from *Aspergillus* sp. As#6 and As#7 strains, an increase occurred in the Km values obtained for the casein substrate. Km value increased from 4.5 to 5 mg mL^−1^ for As#6 strain protease and from 2.5 to 2.6 mg mL^−1^ for As#7 strain protease.^[^ ^33^ ^]^

#### 2.2.4 Reusability

Reusability is one of the most important advantages of immobilization for the enzyme. From an economic point of view, it is quite necessary to control the reusability of the biocatalyst used in industrial applications.^[^ ^34^ ^]^ In the reusability study of immobilized strawberry protease, the activity of the same beads was measured six times, consecutively, and the results are given in Table S2, Supporting Information. It was observed that 62% of the activity was still retained in the third use of the immobilized beads, while there was a serious decrease in the activity after the fourth use. In the immobilization of strawberry protease in Ca‐alginate beads, the reusability remained at an average level, suggesting that the enzyme escaped from the pores of the carrier beads as the number of uses of the immobilized enzyme increased. The similarity between the reusability results of DPP IV serine protease immobilized in Ca‐alginate beads and strawberry protease reusability results may support this idea.^[^ ^24^ ^]^ However, the reusability feature of visceral protease purified from *Labeo rohita* and immobilized in Ca‐alginate beads appears to be much lower than strawberry protease. No activity was observed at the end of the fourth use, while the activity of the enzyme decreased to 25% of the initial activity at the third use.^[^ ^35^ ^]^

#### 2.2.5 Storage Stability

**Figure 2:**
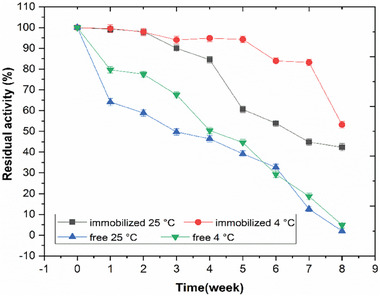
Storage stability graph of immobilized and free protease. Error bars represent the standard deviation of triple measurements. A one‐way ANOVA test followed by the Bonferroni post hoc test was used for statistical evaluation. *n*: 32, *p* < 0.01. The immobilization process significantly increased the stability of the enzyme, and the increase in stability was significant compared to the free enzyme at 4 °C.

To determine the storage stability of the immobilized beads containing strawberry protease, the beads were stored at two different temperatures (4 and 25 °C) and their activity values were recorded for 8 weeks. In addition, to understand how the immobilization process affected the storage stability of the enzyme, the free enzyme was also stored under the same conditions, and its activity was recorded at the same regular intervals. The activity was measured under standard conditions. The storage stability results are given in **Figure** **2**.

Immobilized beads stored at 4 °C retained their activity to a great extent for 7 weeks. At the end of the 7 weeks, the beads still had 83% of their initial activity. At the end of the 8 weeks, it was determined that there was a 46.8% loss in the activity of the beads. Free enzyme stored at 4 °C lost half of its activity at the end of the 4 weeks, and a rapid decrease in activity was observed after the 5th week. After a total period of 8 weeks, the free enzyme retained only 4.9% of its initial activity.

At the end of the 4 weeks, only a 15.5% loss of initial activity was observed in the immobilized beads stored at 25 °C. A more significant decrease in activity occurred during the 4‐ to 8‐week storage period. There was a 46.1% decrease in the initial activity of the beads at the end of the 6 weeks, and a 57.7% decrease at the end of the 8 weeks. The most significant decrease in initial activity occurred in free enzyme stored at 25 °C. At the end of 3 weeks, the free enzyme had lost half of its activity. At the end of the total 8‐week period, the free enzyme had lost almost all of its activity, while at the end of the 6 weeks, a 67.3% loss of enzyme activity was observed.

In general, when the storage stability results of strawberry protease were examined, it can be said that both free and immobilized enzymes were more stable at 4 °C than at 25 °C, and the immobilization process significantly increased the stability of the enzyme (*p* < 0.01 [one‐way ANOVA test], and according to Bonferroni test, after immobilization, the increase in stability was significant compared to the free enzyme at 4 °C). In the study carried out by Su et al., an alkaline protease was immobilized on graphene oxide (GO) nanosheets. Both free and immobilized enzymes were stored in pH 7 phosphate buffer at 4 °C, and their activities were measured regularly every 5 days. According to the results, it was noted that after 25 days of storage, the free enzyme retained approximately 73% of its initial activity, while the GO‐immobilized enzyme retained approximately 85%. Immobilization appeared to increase the stability of the enzyme.^[^ ^36^ ^]^ In another study, the storage stability of acid protease immobilized on functionalized mesoporous activated carbon particles in pH 7 phosphate buffer was investigated at 4 °C. After 5 weeks of storage, 18% of the free enzyme activity and only 50% of the immobilized enzyme activity was retained.^[^ ^37^ ^]^ It can be concluded that immobilized strawberry protease is more stable than both enzymes at 4 °C.

### 2.3 Investigation Results of the Use of Strawberry Protease as a Detergent Additive

#### 2.3.1 Effect of Surfactants, Oxidizing Agents, and Detergents on Enzyme Stability

A good detergent enzyme that is effective during washing must be compatible and stable with commonly used detergent compounds such as surfactants, oxidizing agents, and other additives that may be present in the detergent formulation. Therefore, to investigate the suitability of strawberry protease as a detergent additive, first of all, the crude enzyme (free and immobilized) was treated with various surfactants and oxidizing agents, and the change in its activity was investigated. The residual enzyme activities (%) are given in **Table** **1**.

**Table 1:** Effect of surfactants and oxidizing agents on the stability of strawberry protease.

Detergent components	Concentration [%]	Residual activity [%]^*^		
		FE^#^	IE^#^	IE‐Co^2+^ ^#^
**Control**		**100**	**100**	**100**
**Surfactants**				
SDS	0.5	97 ± 1.0	98 ± 1.1	100 ± 0.06
Triton X‐100	1	90 ± 0.4	92 ± 0.5	97 ± 0.6
Tween 20	1	90 ± 0.5	90 ± 0.8	100 ± 0.7
Tween 80	1	96 ± 0.4	97 ± 0.5	100 ± 0.4
Brij 35	0.1	98 ± 0.8	98 ± 0.6	99 ± 0.8
**Oxidizing agents**				
H_2_O_2_	0.5	53 ± 0.7	54 ± 0.9	56 ± 0.8
NaBO_3_	0.2	89 ± 0.6	100 ± 0.8	100 ± 0.3

* ± Values represent the standard deviation of triple measurements;

FE, free enzyme; IE, immobilized enzyme; IE‐Co^2+^, immobilized enzyme‐Co^2+^;

^#^ *n*: 21, One‐way ANOVA test, *p* > 0.05;

FE, IE, and IE‐Co^2+^ showed similar effects with surfactants and oxidizing agents.

Strawberry protease was determined to be quite stable in the presence of nonionic surfactants. The activities of both free and immobilized strawberry proteases were almost not changed in the presence of Brij 35 compared to the control. After 1 h incubation at 40 °C in the presence of Tween 20, a 10.28% decrease in the activity of the free enzyme occurred. It was observed that this activity loss decreased slightly with the immobilization of the enzyme. It was determined that there was no loss in enzyme activity by immobilizing Co^2+^ ions together with the enzyme. As a result of the stimulating effect of Co^2+^ ions on strawberry protease, the stability of the enzyme to surfactant was increased. The results obtained for Tween 80 and Triton X‐100 were very similar to the results obtained for Tween 20 (this stability increase, which occurred as a result of immobilization [with or without Co^2+^ ions], was not found statistically significant [*p* > 0.05, one‐way ANOVA]).

Free and immobilized strawberry protease enzymes were able to retain most of their activity in the presence of SDS, a highly potent anionic surfactant. The negligible decrease in enzyme activity observed was eliminated by immobilizing Co^2+^ ions together with the enzyme. This is an important finding, as many enzymes are inhibited in the presence of SDS.^[^ ^38^ ^]^

Crude strawberry protease showed different behavior towards the oxidizing agents hydrogen peroxide (H_2_O_2_) and sodium perborate (NaBO_3_). In the presence of NaBO_3_, an inhibition of 11.45% occurred in the activity of free protease compared to the control, while no decrease in the activity of the immobilized enzyme was observed. However, in the presence of hydrogen peroxide, the activity fell by half for all enzyme samples, although slight differences were observed (there is no statistically significant difference [*p* > 0.05] between the results obtained with free enzyme and immobilized enzymes [with or without Co^2+^ ions]).

**Figure 3:**
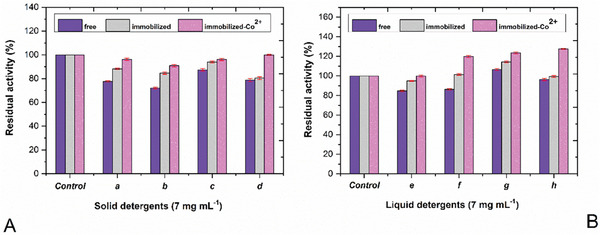
Effect of commercial solid and liquid detergents on strawberry protease activity. Error bars represent the standard deviation of triple measurements. One‐way ANOVA test followed by the Bonferroni post hoc test was used for statistical evaluation. A) Effect of commercial solid detergents on protease activity. *n*: 12, *p* < 0.01. The stability of the enzyme immobilized with Co^2+^ ions in the presence of solid detergents was significantly different compared to the free enzyme. B) Effect of commercial liquid detergents on protease activity. *n*: 12, *p* < 0.03. The stability of the enzyme immobilized with Co^2+^ ions in the presence of liquid detergents was significantly different compared to the free enzyme.

Apart from these, the effect of some commercial detergents on strawberry protease activity was also investigated. The stability results in the presence of *a*, *b*, *c*, and *d* solid detergents and *e*, *f*, *g*, and *h* liquid detergents are given in **Figure** **3**. The chemical composition of commercial detergents *a, b, c, d, e, f, g*, and *h* is given in Table S3, Supporting Information. All tested solid detergents had an inhibitory effect on strawberry protease. This effect was most evident in the free enzyme. The immobilization process increased the stability of the enzyme, just like in detergent additives. Similarly, since the presence of Co^2+^ ions stimulated the enzyme, increasing its stability even higher (*p* < 0.01 [one‐way ANOVA test], and according to Bonferroni test results, the stability of the enzyme immobilized with Co^2+^ ions in the presence of solid detergents was significantly different compared to the free enzyme). Similar results were obtained for *a*, *b*, and *c* detergents. It was observed that the inhibition effect of *d* solid detergent on free and immobilized strawberry protease activity completely disappeared with the immobilization of the enzyme with Co^2+^ ions.

The results of the tests performed with liquid detergents were different from solids. Liquid detergents *e*, *f*, and *h* had low inhibition on free protease. When liquid *e* detergent was used, the inhibition effect on the immobilized enzyme was found to be only 5%, and no inhibition was observed on the enzyme immobilized with Co^2+^ ions. While no inhibition was observed on the immobilized enzyme in the presence of liquid ***h*** detergent, there was an increase in the activity of the enzyme immobilized with Co^2+^ ions. *f* and *g* liquid detergents were found to activate both immobilized enzyme samples. Among these detergents, only *g* activated both free and immobilized enzymes. The partial loss of enzyme activity in the presence of certain detergents can be attributed to the inhibitory effect caused by the components of these detergents. Besides, some components of detergents can also act as activators for the enzyme. As a result, an increase in enzyme activity is observed in the presence of these detergents compared to control.^[^ ^39^ ^]^ According to the results, it is clear that commercial detergents inhibited the enzyme slightly, and immobilization greatly minimized this effect. In addition, Co^2+^ ions stimulated the protease, further increasing its stability (*p* < 0.03 [one‐way ANOVA test], and according to Bonferroni correction, the stability of the enzyme immobilized with Co^2+^ ions in the presence of liquid detergents was significantly different compared to the free enzyme). When the results of the liquid detergents are examined, it is thought that there could be some substances that can activate the enzyme in the formulations of these detergents, and this activation was reflected in the results by combining with the stimulation effect of Co^2+^ ions.

As a result, it was observed that strawberry protease exhibited high stability against all tested oxidants, surfactants, and detergents except hydrogen peroxide. It can be said that enzyme samples were highly sensitive to hydrogen peroxide.

According to the results of the study performed by Sinha and Khare, *Bacillus sp*. EMB9 protease was found to be highly stable in the presence of SDS, Tween 80, and Triton‐X 100, like strawberry protease. The study also reported that EMB9 protease was compatible with various commercial detergents such as Ariel.^[^ ^40^ ^]^ Similarly, Alzwiprase, a serine protease purified from *B. subtilis* DM‐04, was reported to have high stability in the presence of SDS, Triton‐X 100, Tween 20, and Tween 80.^[^ ^39^ ^]^ In another study, the stability of the crude protease obtained from *P. aeruginosa* was tested in the presence of commercial detergents such as Ariel, Persil, and Perwoll. It was reported that the crude protease remained stable in the presence of Perwoll after 1 h of incubation at 30 °C, and apparent losses occurred in the presence of Persil and Ariel.^[^ ^41^ ^]^

The stability of protease enzymes purified from two separate plant sources, *Phalaris minor* seeds^[^ ^42^ ^]^ and *Artocarpus heterophyllus* seeds,^[^ ^43^ ^]^ in the presence of various surfactants, was examined. The effect of Triton‐X100, SDS, Tween‐20, and Tween‐80 surfactants on *Phalaris minor* seed protease activity at different concentration levels (0.1%, 0.5%, and 1%) was tested. According to the results, it was determined that the enzyme maintained its stability at all concentration values in the presence of Triton‐X 100 and Tween‐20, whereas the stability decreased significantly at 1% concentration of SDS and Tween‐80.^[^ ^42^ ^]^ The stability of purified *Artocarpus heterophyllus* seed protease was monitored in the presence of Triton‐X 100, Tween‐80, and SDS surfactants at the same concentrations. It was reported that while the enzyme considerably retained its activity in the presence of Triton‐X 100 and Tween‐80, it almost completely lost its activity in the presence of SDS.^[^ ^43^ ^]^ Strawberry protease appears to be more stable than both enzymes in the presence of surfactants. A study by Mahakhan et al. examined the stability of *B. gibsonii* 6BS15‐4 alkaline protease in the presence of oxidizing agents and found that this enzyme gave similar results to strawberry protease in the presence of NaBO_3_, but was much more stable in the presence of hydrogen peroxide.^[^ ^44^ ^]^ Again, *Haloferax* sp. strain GUBF 2 protease also appears to be more stable than strawberry protease in the presence of hydrogen peroxide.^[^ ^45^ ^]^ The stability of *Halobacillus blutaparonensis* strain M9 serine peptidase against hydrogen peroxide was similar to that of strawberry protease. In addition, strawberry protease was more stable to surfactants Triton‐X 100, Tween‐20, and Tween‐80.^[^ ^46^ ^]^ In the study carried out by Kokwe et al., the effect of various solid laundry detergents (7 mg/mL), including Sunlight, Omo, Ariel, Maq, Surf, Sky, Freshwave, Evaklin, and Prowash, on the activity of the thermoactive *Bacillus* sp. NFH5 metallo‐keratinase was examined. It is observed that the enzyme maintained its stability very well in the presence of commercial detergents, similar to strawberry protease.^[^ ^47^ ^]^ In a study examining the potential of *Bacillus cereus* YQ15 keratinase as a detergent additive, researchers investigated the effects of different commercial detergents known in the local market on enzyme activity. According to the reported results, the residual activity of the enzyme as a result of incubation with seven different detergents separately at 50 °C for 30 min varied by 60% and above.^[^ ^48^ ^]^

### 2.4 Evaluation of Washing Performance

Detergent proteases work best by hydrolyzing large, insoluble proteins in the wash solution. Proteins are initially removed from the fabric surface with detergent components or with water alone. Depending on their size, the pieces separated from the fabric either dissolve in the wash solution or leave themselves back in the fabric. In this process, enzymes used in detergent formulation contribute to detergent effectiveness with the benefits of better stain removal and redeposition prevention thanks to substrate hydrolysis.^[^ ^38^ ^]^

**Figure 4:**
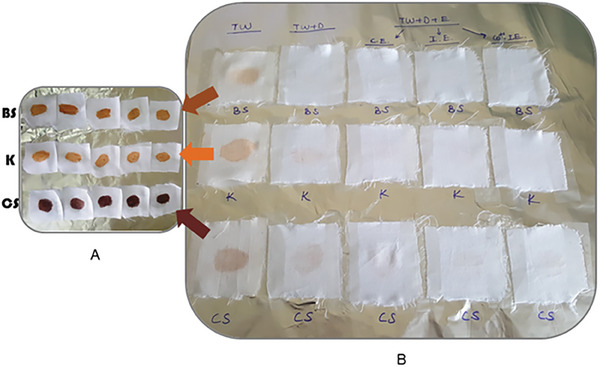
A) Cotton fabric pieces (5 cm × 5 cm) stained with barbecue sauce (BS), ketchup (K), and chocolate sauce (CS). B) Results after washing (in all wash water samples) and drying of cotton fabric pieces stained with barbecue sauce, ketchup, and chocolate sauce. TW, tap water; TW+D, tap water with detergent; TW+D+E, tap water with detergent and enzyme; C.E., crude enzyme; I.E., immobilized enzyme; Co^2+^‐I.E., immobilized enzyme with Co^2+^ ions; BS, barbecue sauce; K, ketchup; CS, chocolate sauce.

The washing performance results indicated that strawberry crude protease could effectively clean various stains such as ketchup, chocolate sauce, and barbecue sauce. Looking at the results in **Figure** **4**, only a slight brightening in the color of the stains was observed on the stained fabrics washed with water; the stains could not be removed from the fabrics. It was observed that when detergent was added to the wash water, each stain was faded but not completely cleaned. The detergent removed most of the barbecue sauce from the fabric; in the last case, there was a slight stain on the fabric. However, most of the stains created by ketchup and chocolate sauce were still clearly visible on the fabric after washing with detergent and water. Very good results were obtained when the enzyme was also added to the wash solution. It is seen that the wash solution containing free crude protease completely cleaned the barbecue sauce stain from the fabric and almost all the ketchup and chocolate sauce stains that the detergent could not remove. It was determined that all stains were effectively (even completely) cleaned from the fabrics when immobilized protease and protease immobilized with Co^2+^ ions were added to the wash solution. Since the immobilization process increased the stability of the crude protease, better results were obtained with the immobilized enzymes compared to the free enzyme. It seems that with the force of immobilization, the enzyme was able to work more effectively when it was less affected by ambient conditions, and even without the stimulation effect of Co^2+^ ions, the immobilized crude protease showed a superior additive feature in cleaning stubborn stains. In general, in the washing process, protease hydrolyzed large insoluble protein fragments that strongly adhered to the fabric, increasing the effectiveness of the detergent

In the study performed by Jellouli et al., the washing performance of *Bacillus licheniformis* MP1 serine protease was evaluated for blood stain removal. As a result, it was observed that the wash solution containing the enzyme and tap water significantly reduced the appearance of blood stains, while the addition of detergent to the wash solution completely removed the blood stains from cotton fabrics.^[^ ^49^ ^]^ In another washing performance study performed with *B. licheniformis* NH1 crude extract containing alkaline proteases, the crude enzyme was tested to remove blood, chocolate sauce, and barbecue sauce stains from cotton fabrics. The fabrics were treated separately with detergent water and detergent water containing the crude enzyme, and as a result, it was determined that the wash solution containing the enzyme was much more effective in removing the stains than the wash solution without the enzyme. When comparing the visual test results of the crude *B. licheniformis* NH1 extract and the crude strawberry protease, it can be said that strawberry protease was more effective in removing stains.^[^ ^38^ ^]^ In a study that is very similar to the strawberry protease considering the tested parameters in terms of suitability for detergent formulations, the success of *B. swezeyi* B2 strain protease in removing chocolate stains was also determined by the wash test. Although the visibility of the stains decreased at the end of the test, they were not eliminated. In this respect, it can be said that the washing performance of strawberry protease is superior to this enzyme.^[^ ^50^ ^]^ In a study where the effectiveness of *B. licheniformis* NWMCC0046 protease in removing different stains such as tea, coffee, coke, milk tea, chocolate, sesame paste, and blood was observed, the washing performance was examined at 20 °C. It was observed that the protease added to the detergent was found to be very successful on six stains except blood, even at a temperature as low as 20 °C.^[^ ^51^ ^]^

## 3 Conclusion

In this work, the efficacy of strawberry crude protease as a detergent additive was investigated, and its contribution to effective washing performance indicated that it has a high potential for use in the detergent industry. It was determined that the strawberry protease worked in great harmony with the tested detergent components and was quite stable in the presence of commercial detergents. Additionally, this study has important findings in terms of increasing the stain‐removing efficiency of the strawberry protease‐added detergent with the stimulation effect caused by the Co^2+^ ions immobilized with the enzyme. The fact that the immobilized enzyme is used in small amounts and exhibits high efficiency will increase the possibility of this enzyme being preferred in industrial processes.

## 4 Experimental Section

### Investigation of the Effect of Co^2+^ Ions Immobilized with Strawberry Protease on Activity

The knowledge obtained from the previous purification and characterization study of the strawberry protease,^[^ ^18^ ^]^ that the Co^2+^ ions strongly activated the strawberry protease, led to the idea of increasing the activity of the immobilized enzyme by using these ions in immobilization. Accordingly, during the immobilization of the enzyme under optimum conditions, Co^2+^ ions were added to the environment and were entrapped with the enzyme in the beads formed. For this purpose, CoCl_2_ was dissolved in the diluted enzyme solution at a final concentration of 1, 2, and 5 mm, and this mixture was immobilized as described in the optimization section. The protease activity was determined for each immobilized enzyme containing Co^2+^ ions at different concentrations and compared with the activity of immobilized enzymes without Co^2+^ ions. The activity and protein concentrations were determined under standard conditions.

### Investigation on the Use of Strawberry Protease as a Detergent Additive—Effect of Surfactants, Oxidizing Agents, and Detergents on Enzyme Stability

The suitability of the strawberry crude enzyme (free and immobilized) as a detergent additive was determined by testing its stability in the presence of certain surfactants such as SDS, Triton X‐100, Tween 20, Tween 80, and Brij 35, as well as oxidizing agents such as H_2_O_2_ and NaBO_3_. For this purpose, a strawberry crude extract containing protease enzyme (350 U mL^−1^) was incubated at 40 °C for 1 h with the aforementioned additives, and then the residual activity (%) of each sample was determined under standard conditions. The activity of the crude enzyme without any additives that were incubated under similar conditions was accepted as 100%.

In addition, the compatibility of strawberry crude enzyme (free and immobilized) with commercial solid and liquid laundry detergents was also examined. The stability of strawberry protease in the presence of solid detergents ***a***, ***b***, ***c***, and ***d*** and liquid detergents ***e***, ***f***, ***g***, and ***h*** was tested in the study. To create similar conditions to real washing conditions, liquid detergents were diluted 100 times, and solid detergents were diluted to a final concentration of 7 mg mL^−1^ in tap water. The diluted detergents were heated at 80 °C for 30 min before adding the strawberry crude protease to inactivate the endogenous enzymes already present in their formulations. Crude enzyme containing 350 U mL^−1^ protease was added to various detergent solutions diluted in tap water and incubated at 40 °C for 1 h, and then enzyme activities were determined under standard conditions. Finally, the enzyme activity of the detergent‐free control sample incubated under the same conditions was accepted as 100%, and the residual enzyme activity (%) values of the samples incubated with detergent were calculated relative to the control.

### Evaluation of Washing Performance

To evaluate the washing performance of strawberry protease (free and immobilized), first clean cotton cloth pieces (5 cm x 5 cm) were stained with ketchup, chocolate sauce, and barbecue sauce. At this step, 100 µL of ketchup, chocolate sauce, and barbecue sauce was applied to individual fabric pieces and then dried thoroughly for 3 days at room conditions. Next, the stained fabric pieces were washed and rinsed at 40 °C. After drying the fabrics in room conditions, the results were visually examined.

Tap water, tap water with detergent, and tap water with enzyme and detergent were tested separately as wash water. The washing performance study was carried out using ***c*** solid detergent. While preparing wash water with detergent, solid detergent was dissolved in tap water to a final concentration of 7 mg mL^−1^. While preparing the enzyme‐added wash water, the enzyme (40 U mL^−1^) was added after the detergent was dissolved in tap water. Dirty fabric pieces were taken into 100 mL of wash water for the washing process. Each flask containing wash water and fabrics was incubated in a closed orbital shaker at 150 rpm and 40 °C for 1 h. The fabrics were removed, rinsed with water, and dried at the end of incubation. Visual examination of the washed fabrics demonstrated the effect of the strawberry protease enzyme in removing stains.

## Conflict of Interest

The authors declare no conflict of interest.

## Author Contributions

E.H.A.: Conceptualization; methodology; validation; investigation; writing—original draft preparation; writing—review and editing. G.A.: Resources; supervision; project administration; funding acquisition.

## Supporting information

Supporting Information

## Data Availability

The data that support the findings of this study are available in the supplementary material of this article.

## References

[1] R. Morellon‐Sterling , H. El‐Siar , O. L. Tavano , Á. Berenguer‐Murcia , R. Fernández‐Lafuente , Int. J. Biol. Macromol. 2020, 162, 394. 32574740 10.1016/j.ijbiomac.2020.06.144

[2] N. Barzkar , Int. J. Biol. Macromol. 2020, 161, 1216. 32534091 10.1016/j.ijbiomac.2020.06.072

[3] K. M. Sharma , R. Kumar , S. Panwar , A. Kumar , J. Genet. Eng. Biotechnol. 2017, 15, 115. 30647648 10.1016/j.jgeb.2017.02.001 PMC6296574

[4] P. Gurumallesh , K. Alagu , B. Ramakrishnan , S. Muthusamy , Int. J. Biol. Macromol. 2019, 128, 254. 30664968 10.1016/j.ijbiomac.2019.01.081

[5] S. E. Barberis , E. Quiroga , C. S. Barcia , C. Liggieri , Electron J. Biotechnol. 2013, 16, 1.

[6] L. Feijoo‐Siota , T. G. Villa , Food Bioprocess Technol. 2011, 4, 1066.

[7] Y. R. Maghraby , R. M. El‐Shabasy , A. H. Ibrahim , H. M. E.l‐S. Azzazy , ACS Omega 2023, 8, 5184. 36816672 10.1021/acsomega.2c07560 PMC9933091

[8] J. S. Bond , J. Biol. Chem. 2019, 294, 1643. 30710012 10.1074/jbc.TM118.004156 PMC6364759

[9] N. Abdullah Al‐Dhabi , G. Ali Esmail , A.‐K. Mohammed Ghilan , M. Valan Arasu , V. Duraipandiyan , K. Ponmurugan , J. King Saud Univ. Sci. 2020, 32, 1258.

[10] L. Vojcic , C. Pitzler , G. Körfer , F. Jakob , Ronny Martinez , K.‐H. Maurer , U. Schwaneberg , New Biotechnol. 2015, 32, 629. 10.1016/j.nbt.2014.12.010 25579194

[11] M. I. El‐Khonezy , E. W. Elgammal , E. F. Ahmed , A. M. Abd‐Elaziz , Biocatal. Agric. Biotechnol. 2021, 35, 102046.

[12] J. G. Dos Santos Aguilar , H. H. Sato , Food Res Int 2018, 103, 253. 29389613 10.1016/j.foodres.2017.10.044

[13] K. Cong Cheng , Z. Sheng Khoo , N. Well Lo , W. Jie Tan , N. G. Chemmangattuvalappil , Heliyon 2020, 6, e03861. 32405547 10.1016/j.heliyon.2020.e03861 PMC7210506

[14] P. Chattopadhyay , A. Karthick , K. Jangir , Tenside Surfact. Det. 2018, 55, 162.

[15] P. Nigam , Biomolecules 2013, 3, 597. 24970183 10.3390/biom3030597 PMC4030947

[16] M. Gagaoua , A. L. Dib , N. Lakhdara , M. Lamri , C. Botinestean , J. M. Lorenzo , Curr. Opin. Food Sci. 2021, 38, 177.

[17] Y. Chen , J. Zhu , C. Zhang , X. Kong , Y. Hua , Food Chem. 2021, 353, 129519. 33740507 10.1016/j.foodchem.2021.129519

[18] E. H. Alici , G. Arabaci , Int. J. Biol. Macromol. 2018, 114, 1295. 29601882 10.1016/j.ijbiomac.2018.03.165

[19] G. Dey , S. Bhupinder , R. Banerjee , Braz. Arch. Biol. Techn. 2003, 46, 167.

[20] A. M. Farag , M. A. Hassan , Enzyme Microbial. Technol. 2004, 34, 85.

[21] B. Doumèche , M. Heinemann , J. Büchs , W. Hartmeier , M. B. Ansorge‐Schumacher , J. Mol. Catalysis B. Enz. 2002, 18, 19.

[22] M. Elibol , A. R. Moreira , Process Biochem. 2003, 38, 1445.

[23] A. Anwar , S. A. Ul Qader , A. Raiz , S. Iqbal , A. Azhar , World Appl. Sci. 2009, 7, 1281.

[24] A. Mittal , S. Khurana , H. Singh , R. C. Kamboj , Enzyme Microb. Technol. 2005, 37, 318.

[25] E. Quiroga , C. O. Illanes , N. A. Ochoa , S. Barberis , Process Biochem. 2011, 46, 1029.

[26] S. Gaur , N. Wadhwa , Afr. J. Biotechnol. 2008, 7, 4602.

[27] R. F. Parrish , K. L. Polakoski , J. Reprod. Fertil. 1981, 62, 417. 7019426 10.1530/jrf.0.0620417

[28] R. K. Narwal , B. Bhushan , A. Pal , S. P. Malhotra , S. Kumar , V. Saharan , Int. J. Biol. Macromol. 2016, 91, 317. 27174906 10.1016/j.ijbiomac.2016.05.025

[29] D. Norouzian , Iran J. Biotechnol. 2003, 1, 197.

[30] A. Dwevedi , in Enzyme Immobilization, Advances in Industry, Agriculture, Medicine, and the Environment, Springer International Publishing, Switzerland 2016, pp. 21–44.

[31] S. K. Marathe , M. A. Vashistht , A. Prashanth , N. Parveen , S. Chakraborty , S. S. Nair , Genet. Eng. Biotechnol. J. 2018, 16, 39. 10.1016/j.jgeb.2017.10.001 PMC6296584 30647702

[32] M. F. Chaplin , C. Bucke , in Enzyme Technology, Cambridge University Press, New York 1990, pp. 80–136.

[33] N Sharma, S. Tripathi, J. Pharm. Biol. Sci. 2013, 7, 86.

[34] A. Sahu , P. S. Badhe , R. Adivarekar , M. R. Ladole , A. B. Pandit , Biotechnol. Rep. 2016, 12, 13. 10.1016/j.btre.2016.07.002 PMC5361075 28352550

[35] S. Geethanjali , A. Subash , Enzyme Res. 2013, 874050. 23533718 10.1155/2013/874050 PMC3600187

[36] R. Su , P. Shi , M. Zhu , F. Hong , D. Li , Bioresource Technol. 2012, 115, 136. 10.1016/j.biortech.2011.12.085 22244904

[37] A. Ganesh Kumar , S. Swarnalatha , P. Kamatchi , G. Sekaran , Biochem. Eng. J. 2009, 43, 185.

[38] N. Hmidet , N. El‐Hadj Ali , A. Haddar , S. Kanoun , S.‐K. Alya , M. Nasri , Biochem. Eng. J. 2009, 47, 71.

[39] S. K. Rai , A. K. Mukherjee , Biochem. Eng. J. 2010, 48, 173.

[40] R. Sinha , S. K. Khare , Bioresour. Technol. 2013, 145, 357. 23219691 10.1016/j.biortech.2012.11.024

[41] S. Grbavcic , D. Bezbradica , L. Izrael‐Zivkovic ´ , N. Avramovic ´ , N. Milosavic ´ , I. Karadzic ´ , Z. Knezevic ´‐Jugovic , Bioresource Technol. 2011, 102, 11226. 10.1016/j.biortech.2011.09.076 22004595

[42] U. Zaman , K. ur Rehman , S. Ullah Khan , S. Badshah , K. M. Hosny , M. A. Alghamdi , H. K. Hmid , M. Alissa , D. M. Bukhary , E. A. Abdelrahman , Int. J. Biol. Macromol. 2023, 233, 123544. 36754264 10.1016/j.ijbiomac.2023.123544

[43] M. Pandey , K. Hajela , Indian J. Biochem. Biophys. 2023, 60, 690.

[44] P. Mahakhan , P. Apiso , K. Srisunthorn , K. Vichitphan , S. Vichitphan , S. Punyauppa‐Path , J. Sawaengkaew , J. Microbiol. Biotechnol. 2023, 33, 195. 36697226 10.4014/jmb.2210.10007 PMC9998202

[45] S. K. Gaonkar , I. J. Furtado , Arch. Microbiol. 2022, 204, 705. 36374350 10.1007/s00203-022-03286-x

[46] A. F. Santos , T. F. O. Souza , D. M. G. Freire , L. Seldin , M. H. Branquinha , A. L. S. Santos , Microbiology 2021, 90, 124.

[47] L. Kokwe , N. E. Nnolim , L. I. Ezeogu , B. Sithole , U. U. Nwodo , Heliyon 2023, 9, e13635. 36852054 10.1016/j.heliyon.2023.e13635 PMC9957710

[48] R.‐X. Zhang , Z.‐W. Wu , H.‐Y. Cui , Y.‐N. Chai , C.‐W. Hua , P. Wang , L. Li , T.‐Y. Yang , BMC Biotechnol. 2022, 22, 26. 36076195 10.1186/s12896-022-00757-3 PMC9454225

[49] K. Jellouli , O. Ghorbel‐Bellaaj , H. B. Ayed , L. Manni , R. Agrebi , M. Nasri , Process Biochem. 2011, 46, 1248.

[50] M. Elhamdi , S. Ghorbel , N. Hmidet , Curr. Microbiol. 2023, 80, 95. 36737528 10.1007/s00284-022-03156-1

[51] W. Zhou , S. Zeng , J. Yu , J. Xiang , F. Zhang , M. S. Takriff , G. Ding , Z. Ma , X. Zhou , J. Basic Microbiol. 2023, 63, 223. 36538731 10.1002/jobm.202200528

