# Supplementary material for: Strawberry Protease as a Laundry Detergent Additive Candidate: Immobilization, Compatibility Study with Detergent Ingredients, and Washing Performance Test
Source: Glob Chall. 2023 Nov 24;8(1):2300102. doi: 10.1002/gch2.202300102 (PMC10784196; doi:10.1002/gch2.202300102)
Supplement: Supplementary file 1 — Supporting Information [file GCH2-8-2300102-s001.pdf]

Global Challenges

Open Access

Supporting Information

for *Global Challenges.*, DOI 10.1002/gch2.202300102

Strawberry Protease as a Laundry Detergent Additive Candidate: Immobilization, Compatibility Study with Detergent Ingredients, and Washing Performance Test

*Esma Hande Alici and Gulnur Arabaci**

Strawberry protease as a laundry detergent additive candidate: immobilization, compatibility study with detergent ingredients and washing performance test

Esma Hande Alici[a], Gulnur Arabaci[a,*]

[a]*Department of Chemistry, Faculty of Science, Sakarya University, Serdivan-Sakarya 54187, Turkey*

*Corresponding Author: Tel: +90 264 2956048; E-mail: garabaci@sakarya.edu.tr
First Author: Tel: +90 264 2955473, E-mail: ealici@sakarya.edu.tr

Appendix A. Supporting information

Content of supporting information

S1. Experimental Procedures

- **Crude extract preparation, protease activity and protein amount determination**
- **Immobilization of strawberry protease**
- **Optimization of immobilization conditions**
- **Optimization of alginate concentration**
- **Optimization of $CaCl_2$ concentration**
- **Amount of loaded enzyme optimization**
- **Number of beads optimization**
- **Determination of immobilization yield and efficiency**
- **Characterization**
- **Determination of reusability**
- **Storage stability**
- **Statistical analysis**
- **References**

S2. Figures and tables

Figure S1. Optimization results of the immobilization conditions of strawberry protease in Ca-alginate beads. Error bars represent the standard deviation of triple measurements. One-way ANOVA test followed by Bonferroni post hoc test was used for statistical evaluation. The p values were found to be <0.0001 and n:12 for all four parameters tested. **(A)** Effect of alginate concentration on the activity of immobilized strawberry protease. There was a statistically significant difference in each of the results obtained at different alginate concentrations compared to the other. **(B)** Effect of calcium chloride concentration on the activity of immobilized strawberry protease. Increase in the specific activity of the protease up to 3% (w/v) calcium chloride concentration was statistically significant and there was no significant difference between 3% (w/v) and 4% (w/v). **(C)** Effect of enzyme solution dilution rate on the activity of immobilized strawberry protease. While there was no significant difference between the results obtained for enzyme dilution factors 2 and 3, the difference between the results of the other dilution factors was found to be statistically significant. **(D)** Effect of the number of beads on the activity of the immobilized strawberry protease. The increase in specific activity due to the increase in the number of beads used was significant after 0.2 g.

Figure S2. The effect of Co^{2+} ions immobilized with the enzyme on the activity of the immobilized strawberry protease. Error bars represent the standard deviation of triple measurements. One-way ANOVA test followed by Bonferroni post hoc test was used for statistical evaluation. n: 9, $p<0.0001$, the change in activity was significant compared to control for all tested Co^{2+} ion concentrations.

Figure S3. Michaelis-Menten and Lineweaver-Burk plot of immobilized and free strawberry protease for BSA substrate. Error bars represent the standard deviation of triple measurements. One-way ANOVA test was used for statistical evaluation. **(A)** Michaelis-Menten plot of immobilized and free protease for BSA substrate. A relatively insignificant decrease ($p>0.05$, n:6) in Vmax was observed after immobilization. **(B)** Lineweaver-Burk plot of immobilized and free protease for BSA substrate. A significant increase ($p<0.0001$, n:6) in Km was observed after immobilization.

Table S1. Yield, efficiency, and recovery results of the immobilization process.

Table S2. Reusability results of immobilized strawberry protease.

Table S3. Composition of commercial detergents ***a, b, c, d, e, f, g, h*** used in the study.

S1. Experimental Procedures

Crude extract preparation, protease activity and protein amount determination

The preparation of strawberry crude extract and enzyme activity assay of strawberry protease were carried out as described in our previous study. [1] Immobilized beads containing equivalent protein to the free enzyme were used for activity determination of the immobilized enzyme. The protein amount was determined by using the Bradford method. [2]

Immobilization of strawberry protease

Entrapment in Ca-alginate gel beads was used for immobilization of strawberry protease. Firstly, optimum conditions for immobilization of the enzyme were determined and then the crude protease enzyme that was immobilized by creating these conditions was characterized.

Optimization of immobilization conditions

Optimization of alginate concentration

First, 2.5 mL of strawberry crude extract and 2.5 mL of pH 6 phosphate buffer (0.1 M) were mixed. Then, 0.05, 0.1, 0.15 and 0.2 g sodium alginate was dissolved in the enzyme solutions separately; so that the alginate concentration was adjusted to 1, 2, 3 and 4% (w/v), respectively. These mixtures were incubated at 4 °C for about 1 h to remove gas bubbles formed by mixing. The cooled mixtures were dropped separately into the pre-cooled gently stirred 20 mL of 2% (w/v) $CaCl_2$ solutions with the aid of 5 mL syringe (without needle). The Ca-alginate immobilized beads formed in this way that containing the enzyme were ripened by keeping at 4 °C for 2 h, then washed with distilled water. Activity and protein concentration was determined for the immobilized beads and the mixture consisting of filtrate and wash water under standard conditions. The specific activity (U/mg protein) was calculated for each alginate concentration and the results were compared for optimization.

Optimization of $CaCl_2$ concentration

Varying concentrations of calcium chloride solution (1–4%, w/v) were prepared for optimization. Immobilization steps were carried out in the same manner as the previous optimization step ($CaCl_2$ concentration was kept constant at 2%, w/v), and then the specific activity was calculated for each calcium chloride concentration.

Amount of loaded enzyme optimization

In order to examine the effect of the amount of enzyme used in the immobilization process on the immobilization efficiency, the crude enzyme extract was diluted with pH 6 phosphate buffer (0.1 M) in different proportions and used in immobilization (dilution factor (DF) = 1, 2, 3, 4; 10-22 mg protein per mL). Immobilization was carried out in the same way as the previous optimization steps (sodium alginate: 1% (w/v); calcium chloride: 3% (w/v)). Finally, the specific activity values were compared.

Number of beads optimization

So as to determine the most appropriate amount of immobilized enzyme beads to be used in activity measurement, enzyme immobilization was performed under standard conditions (sodium alginate: 2%, w/v; calcium chloride: 3%, w/v; DF: 4) and enzyme activity was measured individually for 0.1, 0.2, 0.3 and 0.4 g of beads. The results were compared by calculating the specific activity values.

Determination of immobilization yield and efficiency

So as to determine the success of the immobilization performed, "immobilization yield", "immobilization efficiency" and "activity recovery" were calculated using the following equations. [3]

Yield (%) = (*immobilised activity*/*starting activity*) × 100

The ‘‘immobilised activity’’ was determined by measuring the total residual enzyme activity remained in the enzyme solution after immobilisation process and by subtracting this activity from the total starting enzyme activity.

The “efficiency” term defines the percentage of bound enzyme activity that is observed in the immobilisate:

Efficiency (%) = (*observed activity*/*immobilised activity*) × 100

Lastly, the activity of the immobilisate was compared to that of the total starting activity of the free enzyme by calculating “activity recovery”:

Activity recovery (%) = (*observed activity*/*starting activity*) × 100

Characterization

Characterization studies include optimum temperature and thermal stability, optimum pH and pH stability, and kinetic constants (Michaelis-Menten constant, Km; maximum velocity, Vmax) determination. All the characterization studies were carried out for both free and immobilized strawberry crude protease as described in our previous study [1]. In kinetic studies, BSA solution was used in the concentration range of 0.015 - 0.38 mM and the enzyme activity was measured under standard conditions.

Determination of reusability

Reusability is an important advantage of the immobilization process. For the purpose of determining reusability, the immobilized enzyme was reacted with BSA under standard conditions and its activity was calculated at the end of the reaction. Later, the enzyme used in this reaction was washed with 0.1 M pH 6 phosphate buffer solution and reacted with BSA under the same conditions, and its activity was calculated at the end of the reaction. This process was repeated consecutively, and the loss of activity was determined for each use.

Storage stability

Storage stability of both free enzyme and immobilized enzyme beads was determined for 8 weeks incubation at 4 °C and 25 °C. Free and immobilized strawberry protease was stored separately in 0.1 M pH 6 phosphate buffer at the indicated temperature values for 8 weeks. The change in activity levels of the stored immobilized enzyme and free enzyme was monitored by activity measurement once a week during the total incubation period. Aliquots were removed from the enzyme preparations stored at the specified temperature values after one week incubation and activity was measured under standard conditions at every turn. The residual activity (%) for both free and immobilized enzyme was calculated according to the initial activity.

Statistical analysis

All experiments were performed in triplicate. All results were expressed as mean ± standard deviation of the measurements. Microsoft Excel version 16.77 was used as Statistical software. Data (comparison of the mean values) was analyzed by one way ANOVA test followed by Bonferroni post hoc analysis (if necessary). P values <0.05 were considered statistically significant.

Supplemental References

[1] E.H. Alici, G. Arabaci, *Int. J. Biol. Macromol.* **2018**, 114, 1295-1304.

[2] M.M. Bradford, *Anal. Biochem.* **1976**, 72, 248-254.

[3] R.A. Sheldon, S. van Pelt, *Chem. Soc. Rev.* **2013**, 42, 6223-6235.

S2. Tables and figures

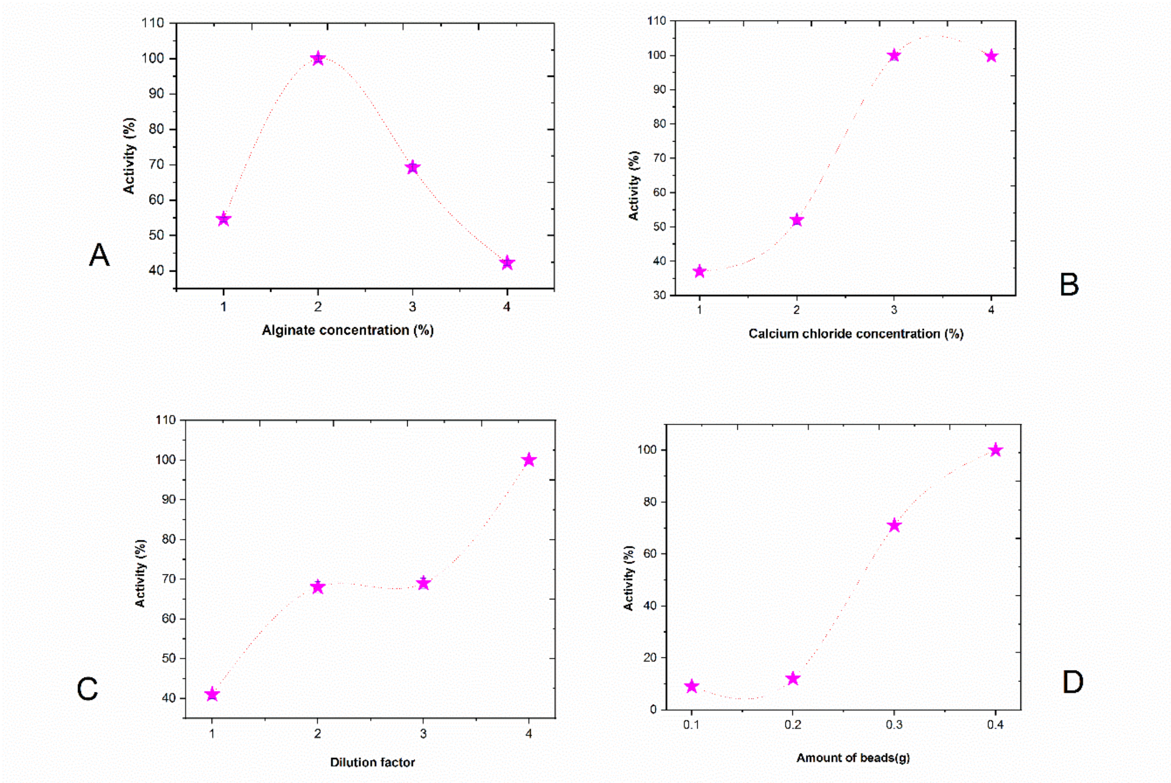

Figure S1. Optimization results of the immobilization conditions of strawberry protease in Ca-alginate beads. Error bars represent the standard deviation of triple measurements. One-way ANOVA test followed by Bonferroni post hoc test was used for statistical evaluation. The p values were found to be <0.0001 and n:12 for all four parameters tested. **(A)** Effect of alginate concentration on the activity of immobilized strawberry protease. There was a statistically significant difference in each of the results obtained at different alginate concentrations compared to the other. **(B)** Effect of calcium chloride concentration on the activity of immobilized strawberry protease. Increase in the specific activity of the protease up to 3% (w/v) calcium chloride concentration was statistically significant and there was no significant difference between 3% (w/v) and 4% (w/v). **(C)** Effect of enzyme solution dilution rate on the activity of immobilized strawberry protease. While there was no significant difference between the results obtained for enzyme dilution factors 2 and 3, the difference between the results of the other dilution factors was found to be statistically significant. **(D)** Effect of the number

of beads on the activity of the immobilized strawberry protease. The increase in specific activity due to the increase in the number of beads used was significant after 0.2 g.

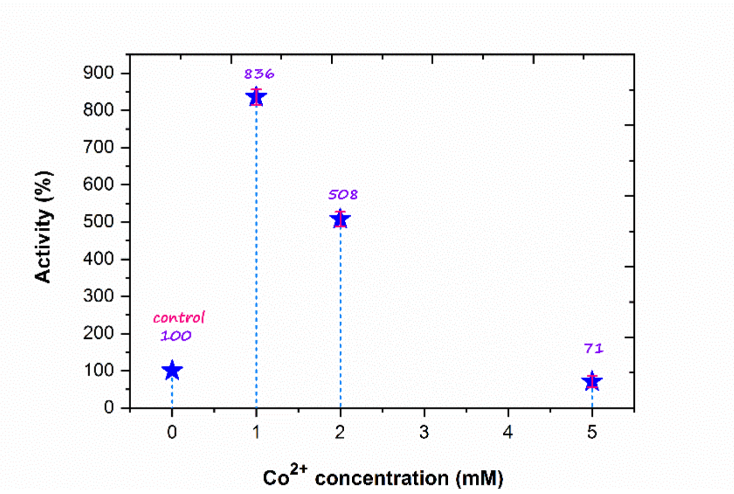

Figure S2. The effect of Co^{2+} ions immobilized with the enzyme on the activity of the immobilized strawberry protease. Error bars represent the standard deviation of triple measurements. One-way ANOVA test followed by Bonferroni post hoc test was used for statistical evaluation. n: 9, $p<0.0001$, the change in activity was significant compared to control for all tested Co^{2+} ion concentrations.

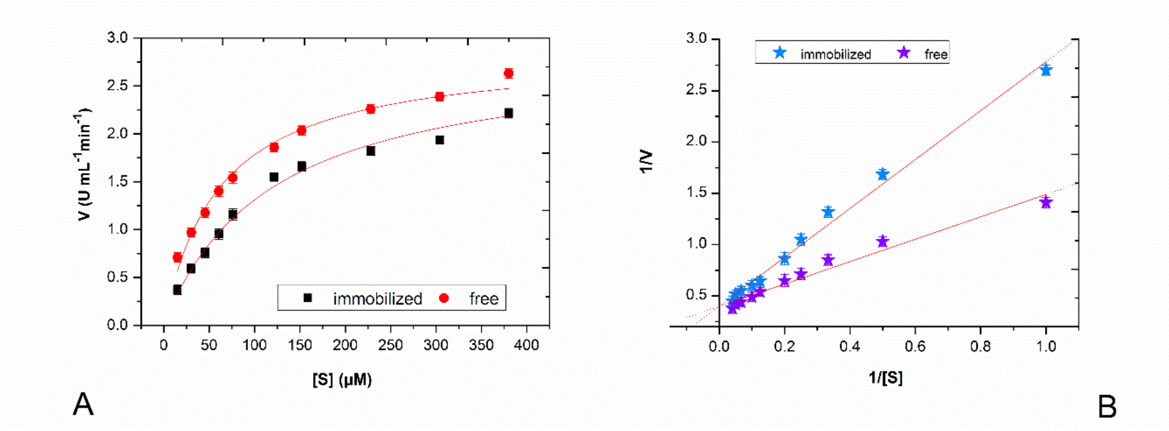

Figure S3. Michaelis-Menten and Lineweaver-Burk plot of immobilized and free strawberry protease for BSA substrate. Error bars represent the standard deviation of triple measurements. One-way ANOVA test was used for statistical evaluation. **(A)** Michaelis-Menten plot of immobilized and free protease for BSA substrate. A relatively insignificant decrease ($p>0.05$, n:6) in Vmax was observed after immobilization. **(B)** Lineweaver-Burk plot of immobilized and free protease for BSA substrate. A significant increase ($p<0.0001$, n:6) in Km was observed after immobilization.

	**İmmobilized enzyme***	**İmmobilized enzyme (with 1 mM Co^{2+})***
**Yield (%)**	65±0.4$^{\#}$	69±0.7$^{\#}$
**Efficiency (%)**	63±0.4$^{\&}$	89±0.3$^{\&}$
**Activity recovery (%)**	41±1.1^{+}	61±0.8^{+}

Table S1. Yield, efficiency, and recovery results of the immobilization process. *± values represent the standard deviation of triple measurements. $^{\#\&+}$One way ANOVA test was used for statistical evaluation and n: 6 (for all three parameters tested). $^{\#}$The increase in yield was found to be statistically insignificant ($p>0.05$). $^{\&+}$The increase in efficiency and activity recovery was statistically significant ($p<0.05$).

**Number of uses**	**Activity (%)***
**1**	100
**2**	86.3±0.39
**3**	61.7±0.57
**4**	31.5±0.55
**5**	20.6±0.73
**6**	17.3±0.89

Table S2. Reusability results of immobilized strawberry protease. *± values represent the standard deviation of triple measurements.

Commercial detergents	Ingredients
a	Sodium Chloride, Sodium Sulfate, Sodium Carbonate, Sodium C10-13 Alkyl Benzenesulfonate, Sodium Silicate, Sodium Carbonate Peroxide, C12-14 Pareth-n, Aqua, TAED, Bentonite, Zeolite, Cellulose Gum, PARFUM, Modified Polycarboxylate, Tetrasodium Etidronate, Brightener 15, Sodium Soap C16-C18 Palm, Sodium Glycolate, Anionic modified polyester, Fatty acids, C16-18 and C18-unsatd., Silicone compound, Sodium polyacrylate, Protease, Polyethylene Glycol, Titanium Dioxide, Microcrystalline Cellulose, Hydrolyzed Corn Starch, Ethoxylated m-toluidine, Hydrated Silica, Dodecylbenzene Sulfonic Acid, Citric Acid, Lipase, Trideceth-n, Magnesium Sulfate, Sucrose, Colorant, Amylase, Mannanase, Kaolin, Sodium Citrate, PEG-33, Chitosan, Acetic acid, Colorant, Hydrochloric Acid, Sodium Thiosulfate, Dichlorodimethylsilane Rx. with Silica, Titanium tetraisopropanolate
b	Sodium Sulfate, Sodium Carbonate, Sodium C10-13 Alkyl Benzenesulfonate, Silicic Acid Sodium Salt, Sodium Polyacrylate, Alcohol Ethoxylate C12-18 7eo, Aqua, Sodium Chloride, Tetrasodium Etidronate, Naal-Silicate Zeolite A, Cellulose Gum, Starch, Perfume Remainder, Disodium 4,4'-Bis[(4-Anilino-6-Morpholino-1,3,5-Triazin-2-Yl) Amino] Stilbene-2,2'-Disulphonate, 7-Octen-2-Ol, 2,6-Dimethyl-, Hexyl Salicylate, 2-T-Butylcyclohexyl Acetate, Butanone, Dodecylthio-Cyclohexenyl, Limonene, 4-Tert-Butylcyclohexyl Acetate, Oxacyclohexadec-12-En-2-One, Protease, Ci 77891, Bjg2-5-15 Pigment Red 57:1 Dispersion~ Bjg2-5-17 Pigment Blue 15:0 Dispersion~ Bjg2-3 1 Pigment Green 7 Disp~ Lipase, Amylase, Cellulase
c	Sodium Sulfate, Sodium Carbonate, Sodium Dodecylbenzene Sulfonate, Sodium Silicate, Sodium Carbonate Peroxide, Zeolite, Sodium Acrylic Acid/Ma Copolymer, Alcohol Ethoxylate 7eo, Tetraacetyl Ethylene Diamine, Perfume, Disodium Anilino morpholino triazinyl aminostilbenesulfonate, Disodium Distyrylbiphenyl Disulfonate, Cellulose Gum, Calcium Sodium Edtmp, Phenylpropyl Ethyl Methicone, Protease, Amylase, Mannanase, Lipase, Water, Ci 74160, Ci 61102
d	Sodium Sulfate, Sodium Carbonate, Sodium Percarbonate, Sodium Linear Alkyl Benzene Sulfonate, Sodium Silicate, Sodium Polyacrylate, Water, C13-C15 Oxoalcohol, Tetraacetylenediamine, Zeolite, Perfume, Hexyl Cinnamal, Polydimethylsiloxane, Anionic Polyester, Diethylene Triamine Penta(Methylene Phosphonic Acid)Disodium Salt, Colored Sodium Sulfate, Colored Sodium Stereat, Carboxymethylcellulose, Amylase, Protease, Mannanase, Lipase, Cellulase, Sulfonated Tetrabenzo-Tetraazoporphyrine, Disodium Distyrylbiphenenyl Disulphonate, Di(Triazinylamino)Stylbene Disulphonic Acid
e	Aqua, Sodium C10-13 Alkyl Benzenesulfonate, Sodium Citrate, C15 Pareth-n, Sodium Palm Kernelate, Sodium Laureth Sulfate, PARFUM, Sodium Cumenesulfonate, MEA-C10-13 Alkyl Benzenesulfonate, Propylene Glycol, Co-polymer of PEG / Vinyl Acetate, Sodium Diethylenetriamine Pentamethylene Phosphonate, Alcohol, Sulfated Ethoxylated Hexamethylenediamine Quaternized, Hydrogenated Castor Oil, C12-14 Pareth-n, PEG/PPG-10/2 Propylheptyl Ether, Glycerin,

	Linalool, Disodium Distyrylbiphenyl Disulfonate, Citric Acid, Sorbitol, Citronellol, Protease, Sodium Formate, Geraniol, Tripropylene Glycol, 2-Aminoethanol Sulfate (Salt), Calcium Chloride, Limonene, Dipropylene Glycol, Ethanolamine, Alpha-Isomethyl Ionone, Phosphodiesterase, Benzisothiazolinone, Amylase, Colorant, Dimethicone, Sodium Chloride, Lyase, Palm Kernel Fatty Acid, Mannanase, Hydroxyethylcellulose, Dimethiconol PEG-2 Stearate
f	Aqua, Sodium Laureth Sulfate, Alcohol Ethoxylate C12-18 7eo, Sodium C10-13 Alkyl Benzenesulfonate, Fatty Acids, C12-18, Sodium Salts, Sodium Citrate, Sodium Chloride, Propylene Glycol, 2-[2-(Dodecyloxy) Ethoxy]Ethanol, Sodium Hydroxide, Glycerin, Sodium Sulfate, Sodium Diethylenetriamine Pentamethylene Phosphonate, Sorbitol, Dipropylene Glycol, Benzene, C10-13-Alkyl Derivs., Cp Methox.Ppg Ma & Methacroylcholine Cl~, Tetramethyl Acetyloctahydronaphthalenes, 3a,4,5,6,7,7a-Hexahydro-4,7-Methano-1h-Inden-5(Or 6)-Yl Acetate, Subtilisin, Disodium Distyrylbiphenyl Disulfonate, Benzyl Acetate, Phenethyl Alcohol, Phenethyl Acetate, Eucalyptol, Peonile, Isobornyl Acetate, Methylundecanal, Hedione, Lınalyl Acetate
g	Aqua, Sodium Dodecylbenzenesulfonate, Sodium Citrate, MEA Dodecylbenzenesulfonate, Sodium Palm Kernelate, C12-14 Pareth-7, C14-15 Pareth-7, Sodium Formate, Sodium Laureth Sulfate, Sodium C12-15 Pareth Sulfate, Sodium Cumenesulfonate, Propylene Glycol, PARFUM, Sulfated Ethoxylated Hexamethylenediamine Quaternized, Hydrogenated Castor Oil, Co-polymer of PEG / Vinyl Acetate, Sodium Diethylenetriamine Pentamethylene Phosphonate, Styrene/Acrylates Copolymer, PEG/PPG-10/2 Propylheptyl Ether, Sodium Hydroxide, Disodium Distyrylbiphenyl Disulfonate, Ethanolamine, Limonene, Protease, Tripropylene Glycol, Polyethylene Glycol, Benzisothiazolinone, Ethoxylated m-toluidine, Calcium Chloride, Sorbitol, Sodium Chloride, Methylisothiazolinone, Dimethicone, Colorant, Hydroxyethylcellulose, Amylase, Dimethiconol, PEG-2 Stearate, PEG-2 Distearate, PEG-6 Palmitate
h	Aqua, Alcohol Ethoxylate C12-18 7eo, Sodium Laureth Sulfate, Sodium C10-13 Alkyl Benzenesulfonate, Sodium Chloride, Fatty Acids, C12-18, Sodium Salts, Sodium Citrate, Sodium Metaborate, Anhydrous, Dipropylene Glycol, Sodium Diethylenetriamine Pentamethylene Phosphonate, Disodium Distyrylbiphenyl Disulfonate, Tetramethyl Acetyloctahydronaphthalenes, Hexahydro-Methanoindenyl Propionate, Hedione, Alpha-Isomethyl Ionone, 3a,4,5,6,7,7a Hexahydro-4,7-Methano-1h-Inden-5(Or 6)-Yl Acetate, Methylisothiazolinone, Benzisothiazolinone, Alpha-Amylase, Lipase, Mannanase, Cellulase

Table S3. Composition of commercial detergents ***a, b, c, d, e, f, g, h*** used in the study.
